# Home used, patient self-managed, brain-computer interface for the management of central neuropathic pain post spinal cord injury: usability study

**DOI:** 10.1186/s12984-019-0588-7

**Published:** 2019-10-30

**Authors:** M. K. H. Al-Taleb, M. Purcell, M. Fraser, N. Petric-Gray, A. Vuckovic

**Affiliations:** 1 Biomedical Engineering Research Division, University of Glasgow, Glasgow, UK; 2 Wasit University, Wasit, Iraq; 3 Queen Elizabeth National Spinal Injuries Unit, Queen Elizabeth University Hospital, Glasgow, UK

**Keywords:** Central neuropathic pain, Neurofeedback, Spinal cord injury, Brain computer Interface, Usability

## Abstract

**Background:**

Central Neuropathic Pain (CNP) is a frequent chronic condition in people with spinal cord injury (SCI). Previously, we showed that using laboratory brain-computer interface (BCI) technology for neurofeedback (NFB) training, it was possible to reduce CNP in people with SCI. In this study, we show results of patient self-managed treatment in their homes with a BCI-NFB using a consumer EEG device.

**Methods:**

*Users*: People with chronic SCI (17 M, 3 F, 50.6 ± 14.1 years old), and CNP ≥4 on a Visual Numerical Scale. *Location*: Laboratory training (up to 4 sessions) followed by home self-managed NFB. *User Activity*: Upregulating the EEG alpha band power by 10% above a threshold and at the same time downregulating the theta and upper beta (20-30 Hz) band power by 10% at electrode location C4. *Technology*: A consumer grade multichannel EEG headset (Epoch, Emotiv, USA), a tablet computer and custom made NFB software. *Evaluation*: EEG analysis, before and after NFB assessment, interviews and questionnaires.

**Results:**

*Effectiveness*: Out of 20 initially assessed participants, 15 took part in the study. Participants used the system for 6.9 ± 5.5 (median 4) weeks. Twelve participants regulated their brainwaves in a frequency specific manner and were most successful upregulating the alpha band power. However they typically upregulated power around their individual alpha peak (7.6 ± 0.8 Hz) that was lower than in people without CNP. The reduction in pain experienced was statistically significant in 12 and clinically significant (greater than 30%) in 8 participants. *Efficiency*: The donning was between 5 and 15 min, and approximately 10–20% of EEG data recorded in the home environment was noise. Participants were mildly stressed when self-administering NFB at home (2.4 on a scale 1–10). *User satisfaction*: Nine participants who completed the final assessment reported a high level of satisfaction (QUESQ, 4.5 ± 0.8), naming effectiveness, ease of use and comfort as main priorities. The main factors influencing frequency of NFB training were: health related issues, free time and pain intensity.

**Conclusion:**

Portable NFB is a feasible solution for home-based self-managed treatment of CNP. Compared to pharmacological treatments, NFB has less side effects and provides users with active control over pain.

**Trial registration:**

GN15NE124, Registered 9th June 2016.

## Background

Primary consequences of Spinal Cord Injury (SCI) include loss or impairment of sensation and voluntary control of muscles. A related secondary consequence of the injury is chronic neuropathic pain. It is believed that neuropathic pain below the level of injury has a central origin, and is therefore also referred to as Central Neuropathic Pain (CNP) [1]. In 40% of SCI patients, pain is severe, persistently interfering with the activities of daily living [2, 3]. As a consequence of this, patient’s sleep quality is reduced associated with high levels of anxiety and depression [4]. A combination of low self-efficacy and pain intensity has been associated with reduced quality of life in people with SCI [5]. The CNP following spinal cord injury not only affects patients’ health status and quality of life but also has an economic impact on the patient and the wider society [4].

Patients with CNP perceive pain as coming from the part of body affected by the injury, but the origin of the pain is actually in the central nervous system. Magnetic resonance imaging studies revealed changes in brain anatomy due to CNP [6]. The activity of the sensory-motor cortex is in particular affected by this type of pain [7]. Several studies defined electroencephalographic (EEG) markers of CNP, such as: reduction of alpha band power and shift of dominant alpha frequency towards lower frequencies, increased theta and beta band power due to thalamo-cortical dysrhythmia, and a reduced ratio between EEG eyes open and eyes closed states [8–11]. Recently, our group identified asymptomatic EEG markers preceding the physical sensation of CNP in people with subacute SCI [12]. Changes in EEG activity may precede the onset of pain, and to some extent may cause pain.

CNP symptoms do not respond well to medications. Drugs used to treat this type of pain are often associated with significant adverse side effects and complete pain relief is rare [13, 14]. A Cochrane study analysis showed that neuromodulatory interventions, which modify brain processes underlying the experience of pain have the potential to relieve pain [15]. These interventions may be used either to complement or to replace pharmacological treatments. The most studied noninvasive neuromodulatory treatments of CNP are repetitive transcranial magnetic stimulation (rTMS) and transcranial direct current stimulation (tDCS) [15]. Both techniques require external electrical or magnetic stimulation of the cortex.

Neurofeedback (NFB) is a neuromodulatory treatment, it enables people to modulate their brain activity at will. It relies on BCI technology, which enables analysis and visualization of EEG signals in real time [16]. Neurofeedback has been used for the treatment of various conditions such as attention deficit hyperactivity disorder, epilepsy, migraine, depression, to name a few [16, 17]. Neurofeedback has also been used for the treatment of chronic pain, such as complex regional pain syndrome [18], fibromyalgia [19], migraine [20] and our group demonstrated its effectivness for treating CNP in people with SCI in controlled clinical conditions [21, 22].

Results from the literature suggest that NFB tunes brain oscillation towards a homeostatic set-point which affords an optimal balance between network flexibility and stability [23]. This hypothesis is relevant in light of CNP, which is considered a consequence of disturbed homeostasis of the sensory system, in particular its thermal pathways [24, 25].

A particularly appealing aspect of NFB, compared to other neuromodulatory treatments which require technology (rTMS, tDCS [15]), is that it does not require an external stimulus apart from visual feedback. It enables patients to actively take part in the treatment, by shifting the locus of control from external to internal [26]. A participant in our previous study commented that “*Previously pain controlled my life, now I’m in control of pain*” [27]. This aspect of NFB is of particular importance in the SCI population, who, due to physical disability, are constantly struggling to get control over their own lives [28].

We performed our previous study [21], in a laboratory within a spinal injuries centre, we used laboratory EEG equipment with a cap, gel, wires and costly licensed software. Patients often had to travel an hour or more to hospital causing fatigue, which was counterproductive to NFB. A quote from one of the participants nicely summaries patient requirements: “*If a handheld or portable device could be made which you could switch on and do the same things that we’ve been doing in the hospital here, then that would be a big advance”* [27].

Results of BCI usability home based studies from the literature [29–31] indicate that home based systems operated by non-experts in uncontrolled conditions must be effective, have a tele-monitoring system, easy to use, portable and be inexpensive. A key point of usability is that users can employ a particular technology with relative ease according to the specific context of use. This ensures that the device not only does its intended purpose but also that it fits around users’ lifestyles. Such devices can only be tested in the home rather than a laboratory environment. This approach should minimise the ‘burden of treatment’ [32] which is a frequent reason for abandonment of much wider used technology such as splints, walkers or scooters [33] .

Brain computer interface usability studies with patients have been tested on several paradigms, including BCI spellers, painting, gaming, environmental control (smart homes) and cognitive rehabilitation [30, 31, 34–43]. However, a very small number of these studies [30, 37, 40] have been performed in patients’ homes, and a trained specialist was often present. A “Back home” project, one of the largest of this kind, tested BCI designed for spelling, gaming and internet browsing on 9 patients at a hospital. They rated speed, ease of use, effectiveness, reliability and comfort as the most important properties of BCI [29].

In a subsequent study, Miralles et al. [30] tested the “Back home” system on a number of patients at hospital but only two patients managed to use the device in their homes for 6 weeks. Daly’s et al. [34] usability study of BCI for cognitive rehabilitation of people with traumatic brain injury initially involved 10 participants but only 5 completed all three sessions. These studies demonstrate challenges of organising studies outside the controlled laboratory or clinical environments.

BCI user-centred design has four stages [44]: understand and specify the context of use, specify the user requirements, produce design solutions and evaluate design against requirements. In this study, we present the last two stages, design solution and end user evaluation. Our proposed design solution is a wearable inexpensive version of BCI NFB. Previously we tested reliability of the BCI NFB presented here in a single session on 18 able-bodied people [45] but we did not test the effect of NFB on pain and participant’s ability to self-manage BCI equipment.

Recently, Rhiu et al. [46] proposed a BCI usability framework that is an adapted version of a usability framework for consumer audio-visual technology [47]. In this study we adopted Rhiu’s framework to test the usability of patient self-managed BCI NFB treatment of CNP using wireless consumer BCI technology. People with SCI affected by CNP range in impairments from those with mild walking difficulty to complete tetraplegia, such people are unable to use their hands and rely on their caregivers for activities of everyday living [2, 3]. This provided the opportunity to test the usability of the system on people with different abilities, within the same BCI NFB paradigm.

The aim of this study is to present a portable BCI NFB solution and to test the usability of the system for home based, self-managed treatment of CNP in people with SCI.

## Materials and methods

**Fig. 1:**
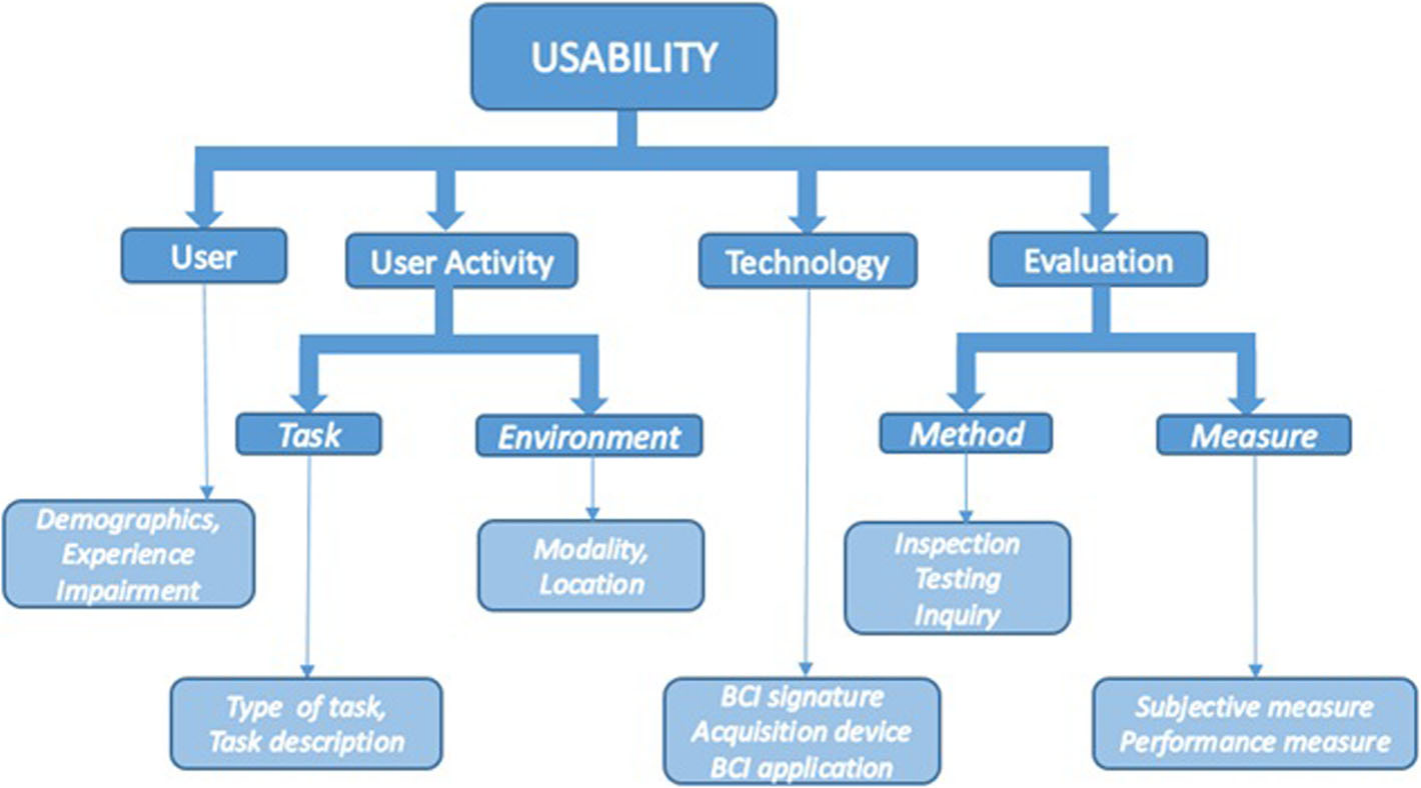
Usability framework (Rhui et al. 2018)

The usability framework [46] consists of 6 components divided into 4 groups, (i) User, (ii) User activity, comprising Task and the Environment, (iii) Technology and (iv) Evaluation, comprising Methods and Measures (Fig. 1). The framework has been recently published, and the examples in [46] were based on previously published papers, which did not follow the framework from the outset. In order to implement the framework, we further subdivided these 6 generic components to suit to the particular study design.

### Users

We expanded component “Users” into three categories: user impairment, user demographics and user experience.

#### User impairment

Twenty people with SCI (17 male and 3 female, age 50.6 ± 14.1 years) participated in this study. They were previously diagnosed with chronic CNP [48]. The American Spinal Injury Association (ASIA) Impairment Classification was used to determine the neurological level of SCI [49]. A SCI is defined by the level of injury and the completeness of injury. The level of injury C (cervical) corresponds to tetraplegia while T (thoracic) and L (lumbar) to paraplegia. The completeness of injury is defined as: A-sensory and motor complete, B-sensory incomplete and motor complete and C and D-sensory and motor incomplete. Typically sensory D incomplete are able to walk often with some form of assistive devices, like canes or foot splints. In this study eight participants were able to walk, 9 were paraplegic wheelchair users who could use their hands while three patients were tetraplegic and could not use their hands.

There were no inclusion restrictions with respect to the level or completeness of the injury, as there is no clear evidence between these factors and the incidence of CNP [2]. Table 1 shows participants’ demographic information.

**Table 1:** Participants’ demographics information

Parti.	Gender	Age	Level of injury	ASIA	Years since SCI	Pain intensity (VNS)	Medication
P1	M	62	L3/L4	D	9	10	Pregabalin
P2	M	51	T6/T7	D	7	7	Gabapentin
P3	F	56	T5	D	3	7	Tramadol
P4	M	64	T4	A	7	6	Tramadol
P5	M	66	L3	D	5	5	/
P6	M	59	C2	B	5	8	Pregabalin
P7	M	59	C2	A	7	5	/
P8	M	50	C3/C5	D	3	5	/
P9	F	54	T5	A	7	5	Pregabalin
P10	M	35	C4	D	15	10	Gabapentin
P11	M	42	C2	A	1	4–5	Gabapentin
P12	M	49	T6	B	1	5	Duloxetine
P13	F	21		D	10	6	/
P14	F	54	C6/C7	A	5	6	/
P15	M	58	T6/T7	A	30	9–10	Nabline
P16	M	18	L3/4	D	7	4	/
P17	M	42	C5-C7	A	15	6	Baclofen/ Pregabalin
P18	M	58	L4	A	21	6	Baclofen/ Pregabalin
P19	M	42	L2	A	13	7	/
P20	M	72	T10	A	1	8	Baclofen

*ASIA* American Spinal Injury Association (ASIA) Impairment Classification

*VNS* Visual Numerical Scale (VNS, 0 = no pain, 10 = worst pain imaginable

The inclusion criteria were: intensity of CNP ≥ 4 on the Visual Numerical Scale (VNS, 0 = no pain, 10 = worst pain imaginable), CNP ongoing for at least 6 months, aged between 18 and 75 years, no self-reported history of brain disease or injury, normal or corrected to normal vision and basic computer skills. The exclusion criteria were: presence of chronic or acute muscular or visceral pain ≥4 VNS, epilepsy, stroke, traumatic brain injury or any other self-reported neurological problem. All participants had below level pain while participants 3, 8 and 12 also had pain at the level of injury. Below level pain has a central origin and is caused by the injury to the spinal cord while at level pain may occur due to the injury to the root or spinal cord, thus it may have central or peripheral origin [50, 51]. Participants typically described the pain sensation as constant burning or freezing, pins and needles, tingling or squeezing combined with the intermittent electrical shock sensations.

Most of the participants were using some types of CNP medications, such as anticonvulsants and antidepressants, which in large doses (larger than normally prescribed) might affect the EEG signal [52]. Participants were asked not to change their medications throughout the study.

#### User demographics

All participants had at least secondary school education. Six participants were employed; six retired, six stopped working after injury and two were students. All participants lived within a two-hour drive of the spinal injuries centre. All three tetraplegic and four paraplegic participants had a caregiver (professional or a family member) who was present during training sessions at the hospital and later assisted with NFB at home.

#### User experience

Two participants took part in our previous study 4 years ago [21] and were familiar with the NFB protocol but had never used BCI outside the laboratory and on their own. Although previous practice might have given them the advantage of learning NFB strategy, we do not believe that after 4 years previous NFB had an effect on their brain activity. The rest of participants were not familiar with the concept of NFB. Half of the participants have previously tried nonpharmacological treatment of CNP, acupuncture or mindfulness, both available through the healthcare system.

### User activity

#### User environment

User environment comprises of “Feedback modality” and “Location”.

##### Feedback modality

Neurofeedback training was provided in a form of visual feedback, showing EEG power in selected frequency bands on a graphical user interface.

##### Location

**Fig. 2:**
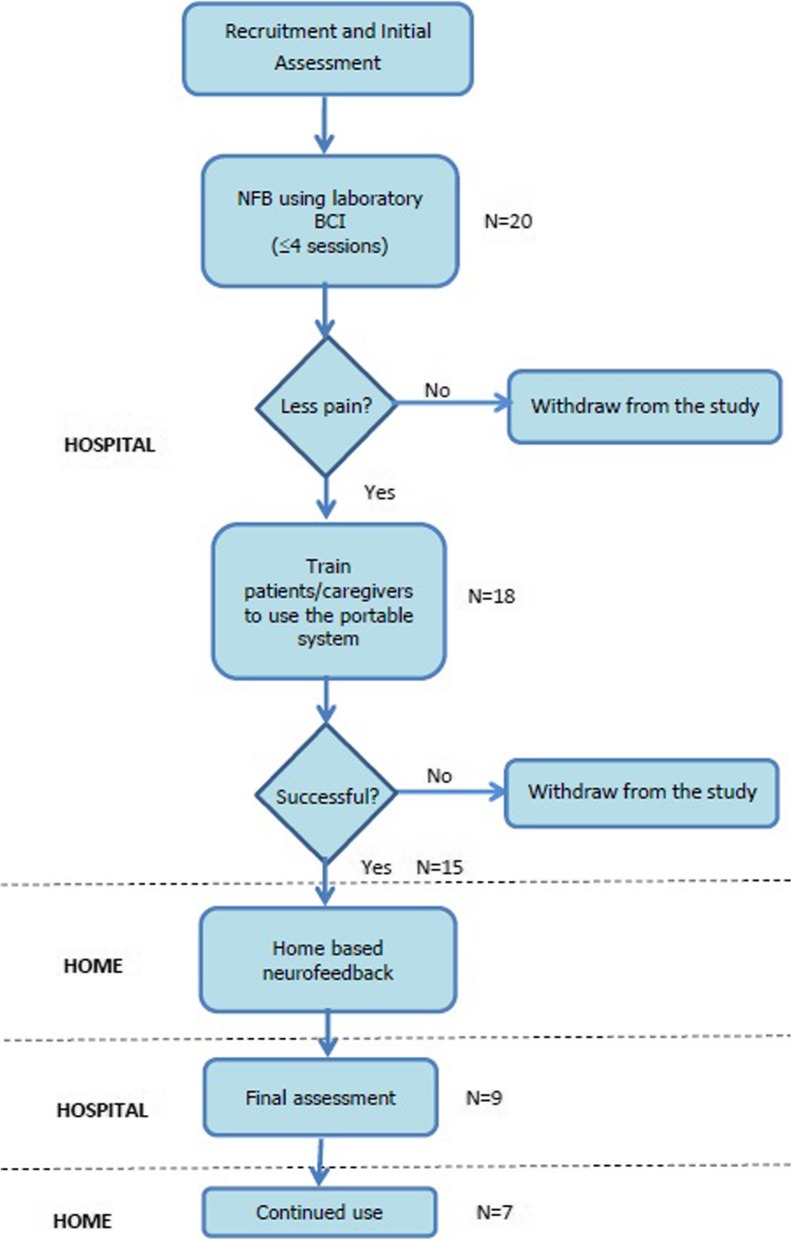
Research protocol. N presents the number of participants involved in each phase

All participants were initially trained at the hospital. After the initial supervised training they used the BCI NFB system in their homes. The experimental protocol is shown in Fig. 2.

#### User task

User task comprised of “Type of the task” and “Description of the Task”.

##### Type of the task

Here we describe the SCI participant’s task only. Rhiu et al. [46] suggested that all BCI tasks should be classified as opened and closed tasks, depending on weather researchers or participants define the outcome of the task. The NFB tasks can be described as closed self-managed task, i.e. the neurofeedback task was set by the researchers (thus is called a closed task) but patients freely defined the strategy.

##### Description of the task

According to Rhiu et al. this section describes the end users task within BCI sessions, and does not include the role of different participants within the research protocol. We modified this section to include all participants. The task of the research group was to perform initial NFB training, assessments and to provide support (in person or tele support) when needed throughout the study. Depending on the level of injury of SCI participants, the task of the caregivers varied from taking notes and photos during training to doing complete NFB software and hardware settings. The task for participants with SCI varied depending on their level of independence. Participants who attended training on their own, also self-managed NFB therapy. Participants with caregivers either performed only NFB without physical contact with a tablet (tetraplegic participants *N* = 3) or self-managed the NFB software while caregivers managed the EEG headset (*N* = 4).

*Research Protocol* consisted of the following steps (Fig. 2):

1. Familiarization with the study: Interested participants (*N* = 20) were invited to the laboratory for a demonstration of the system.
2. Initial assessment and neurofeedback training: This involved practicing NFB using a laboratory EEG device (g.USBamp, Guger Technologies, Austria). Participants with an initial physical response to NFB were identified and offered training on a portable BCI-NF device. The initial assessment of the effect of neurofeedback on pain consisted of up to four NFB sessions. The number of sessions was based on the literature [53], though our results on able-bodied individuals [45] indicated that people can learn the NFB strategy within one thirty minute session. Based on our previous experience [21] we were looking for sensory responses to NFB such as: reduction in pain of at least one point on the VNS, pleasant warmth replacing the sensation of burning or freezing, tingling in the toes or finger tips, wet sensation in the legs. Five participants decided to withdraw before completing all four NFB sessions, two due to lack of response and three could not commit to the study.
3. Patient and caregiver training to use a wearable BCI (*N* = 15); This involved up to four training sessions at the hospital with the Emotiv (Epoch, USA) headset and custom made NFB application. Some of these training sessions were organized on the same day as the initial neurofeedback assessment with g.USBamp, to save patient time. Training involved three steps:
  - Learning to place the headset at the correct location on the head.
  - Learning to correctly moisten the electrodes to achieve low electrode-skin impedance, measured using Emotiv proprietary software.
  - Learning to use the custom-designed software.

Two manuals were provided to patients, the Emotiv proprietary manual and a custom written manual for the NFB application.

**Fig. 3:**
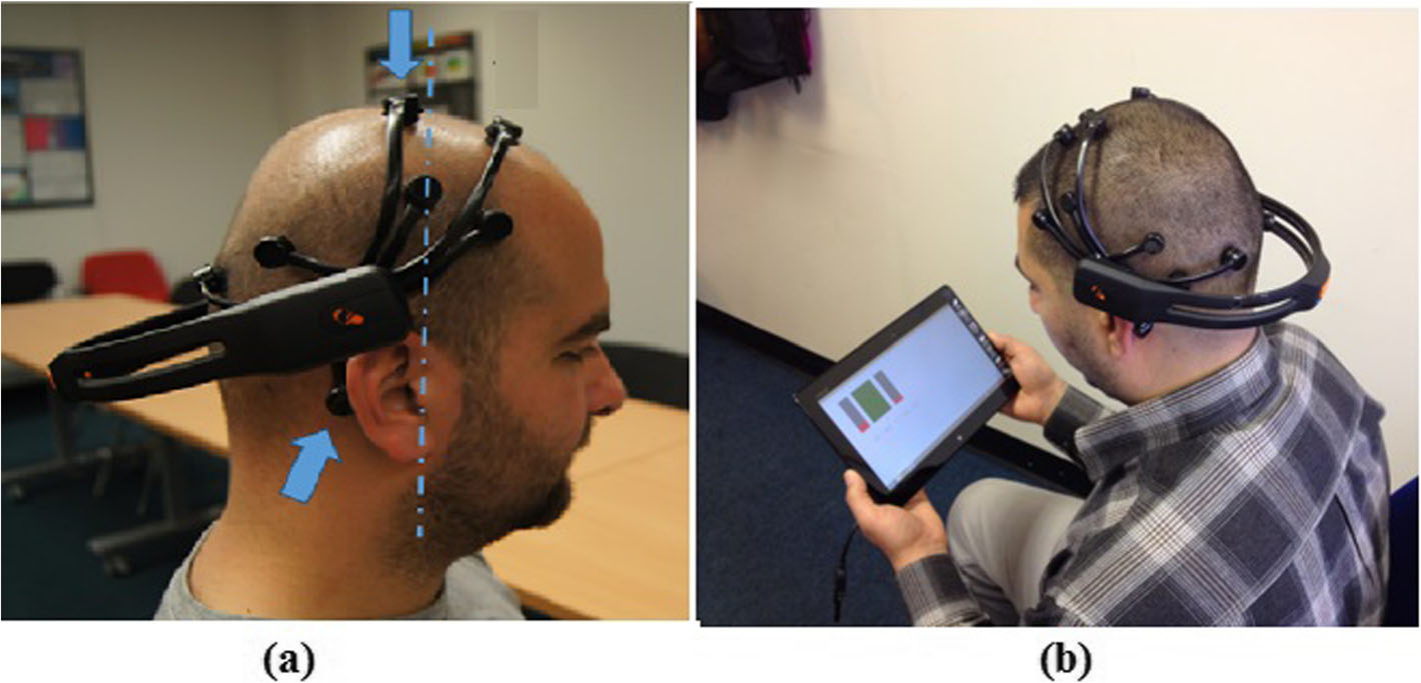
**a** A member of research team demonstrating correct placement of the headset. Long arm EEG electrodes, marked with the arrows were placed over the central cortex. The electrode from which NFB was provided was placed posteriorly with respect to the imagined vertical line (dashed red line in the figure) aligned with participants’ ears. The electrode was placed between electrode location C2 and C4, exact location varies slightly depending on the head size. Image presented in user manual created for patients. **b** BCI NFB system consisting of EEG headset and tablet

4. Home based neurofeedback: Participants were asked to use the Emotiv device in their homes at least once a week over a period of 2 months and were offered the opportunity of keeping the headset and a tablet with NFB software following completion of the study. They all attended a follow up meeting in person at the hospital where they had to demonstrate independent use of BCI NFB and take part in a semi-structured interview. Additional training sessions were organised as required.
  Description of the NFB task: NFB can be classified as a mental task [46]. During NFB, participants were sitting about 1 meter from the computer screen (Fig. 3). They had to self-regulate their brain activity from the area of the primary motor cortex (electrode location C4-C2) using visual feedback. One training session lasted 30 minutes and was divided into 5 minute sub sessions to avoid tiredness. Before NFB training, patient’s baseline EEG activity was recorded for 2 minutes in the relaxed, eyes open state. The task was to increase (upregulate) the alpha band power by increasing the size of the middle bar by more than 10% above the baseline value and to decrease (downregulate) the theta and beta band power by more than 10% with respect to the baseline. Bars were green when related EEG power was in the desired range, otherwise their colour was red. Participants were instructed to “keep bars green”.
5. Final assessment with debriefing: This stage involved questionnaires and EEG data collection from the tablet given to participants to use at home. Alternatively, data was collected remotely via the internet. Data was collected either 2 months after the first use or upon withdrawal from the study (e.g. because of unrelated health conditions, surgical interventions, holidays etc.), whichever happened first. In addition, sample EEG data was collected during a 2 weeks check-up meeting to inspect the quality of recorded data

### Technology

This section describes the acquisition system, BCI signatures and BCI application.

#### Acquisition device

There were two acquisition devices, a laboratory universal 16 channel bio signal amplifier, g.USBamp (Guger Technologies, Austria) and a wearable consumer grade EEG headset Epoch (Emotiv, USA). For the g.USBamp the EEG sampling frequency was 256 samples/s, the right ear served as a reference and the left ear as a ground. The electrode-skin impedance was set to under 5kΩ prior to the EEG recording. EEG signal was filtered between 2 and 30 Hz and additionally notch filtered at 50 Hz using 5th order IIR digital Butterworth filters within the g.USBamp device. Previously developed NFB software [21] was used. The NFB was developed in Simulink, Matlab (Mathworks, USA) and LavView (National Instruments, USA) using rtsBCI software (Guger technology, Austria). The main reason for using usbamp was to test participant’s response to NFB using a higher grade EEG device and to precisely locate electrode C4 [54] using an EEG cap. Following removal of the EEG cap, a mark from the EEG gel remained on the participant head. This was used to position the wearable EEG headset albeit taking a photo of the location of the headset.

Participants used a 14 channel wearable EEG headset (Epoch, Emotiv, CA) for NFB training at home. Sampling frequency was 128 samples/s and two reference electrodes were placed parietal, above the ears (Fig. 3) for CMS/DRL noise cancellation. Wireless communication between the EEG device and tablet, was based on proprietary 2.4GB wireless technology. Impedance was colour coded going from black (no contact) to green, where green colour corresponded approximately to 10 kΩ.

The original electrode layout of the Epoch device does not cover the central cortex. For that reason, the headset had to be tilted back so that two pairs of long handled electrodes were located over the central cortex. The electrode used for NFB was located posterior from the imagined vertical line going through the patients’ ears and was located approximately between the C4 and C2 electrode locations (Fig. 3). During training at the hospital a photo was taken from the side, the back and the top for participants as a reminder for setting up the system at home.

**Fig. 4:**
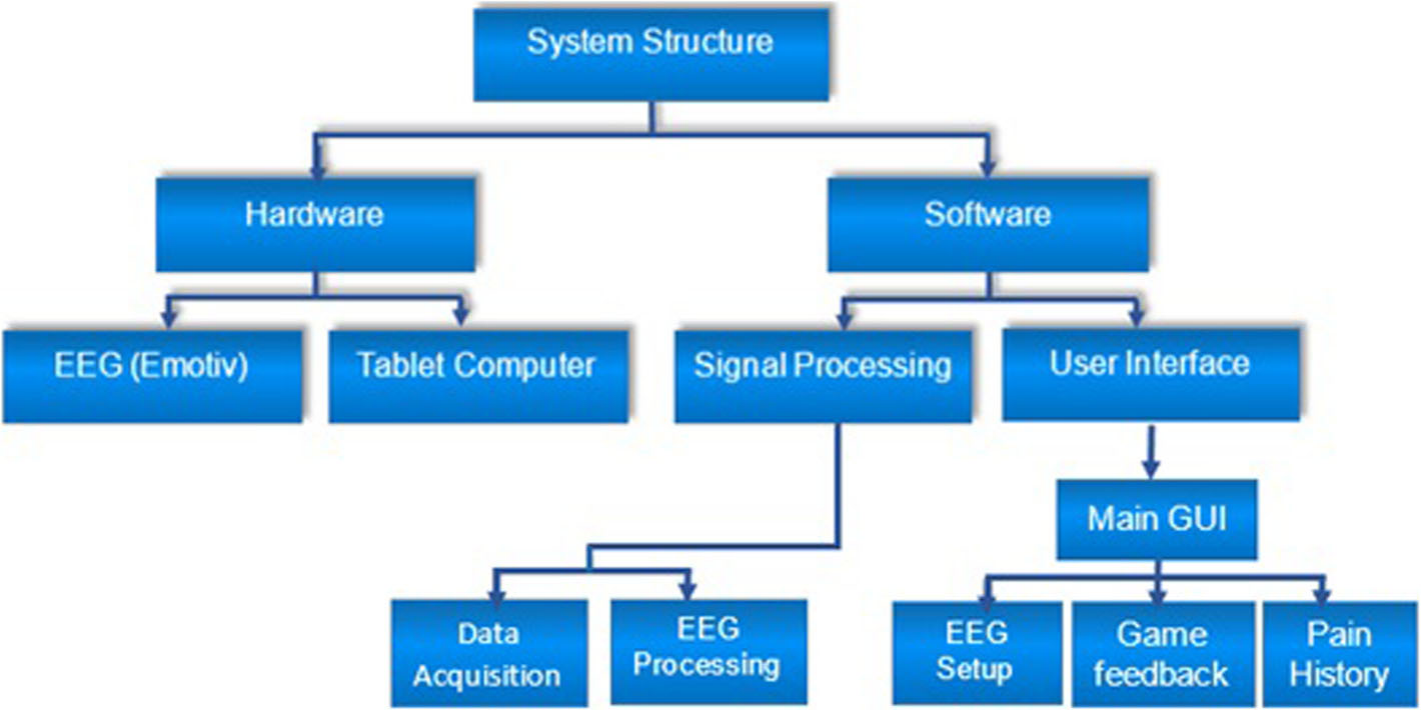
Hardware and software system structure

BCI hardware comprised of: EEG headset, computer tablet and a dongle for wireless communication. Software comprised of Emotiv proprietary software and custom made software. Emotiv proprietary software enabled visualisation of raw EEG and an impedance check. The NFB application comprised of software for signal processing units and Graphical User Interface (GUI) units. The former consisted of data acquisition unit and EEG processing units. The GUI unit consisted of Main GUI screen, which provided further access to EEG setup GUI, pain diary GUI and NFB games GUIs. NFB game GUIs will be described further in the text Fig. 4.

#### BCI signatures

The protocol developed in Simulink and LabView [21] was replicated in C++. This was used to enable a large number of users to use the inexpensive systems at the same time (without providing Matlab and LabView licence) and to use tablet computers. EEG signal was filtered in four frequency bands: 2–30 Hz, theta (4–8 Hz), alpha (9–12 Hz) and higher beta (20–30 Hz) using a 5th order Butterworth filter. Power in each band was calculated over 0.5 s moving average windows and a relative power was calculated by dividing the power of each band (theta, alpha and higher beta) by the EEG power in the 2–30 Hz frequency band. In that way, the EEG power in each frequency band was normalized and expressed as a percentage irrespective of EEG amplitude of an individual user. Relative power during NFB has been constantly compared to the baseline values in corresponding bands. This was reflected by changing colours (from red to green) in the GUI with bars or by changing speed in a GUI with cars.

For the off-line analysis, due to single channel recording, EEG was inspected manually and signal having an amplitude greater than 100 μV or containing EOG was manually removed. On average about 10–20% of EEG signal was removed.

Somewhat higher alpha band (9–12 Hz), without the lowest frequency (8 Hz), was chosen because people with SCI and CNP have on average a lower dominant alpha frequency than able-bodied people as well as people with an SCI with no pain [8–11]. The purpose of this was to increase the dominant alpha frequency through NFB training as well as to increase the alpha band power. Reduced alpha band is regarded as a signature of chronic pain in general [55] and was also reported in people with SCI and CNP.

In parallel participants had to decrease the theta and higher beta band power, that are normally increased in people with CNP due to thalamo-cortical dysrhythmia [8]. Due to dysrhythmia, thalamocortical modules in theta mode exert less collateral inhibition to the neighbouring modules, which are thereby activated in higher beta and gamma frequency ranges. This phenomena is called “the edge effect” [8]. While theta and beta band were related to EEG signatures of pain, these two bands are also related to noise coming from blinking (theta band) and muscle activity (beta band). Thus minimising theta and beta power also minimised the online noise. This is a common strategy in commercial NFB software (e.g. Nexus, Mind Media, the Netherlands).

#### BCI application

According to the classification proposed by Rhiu et al. [46] all applications can be classified into three categories (i) replacing lost communication (ii) supplementing normal function (iii) replacing lost motor function and promoting neuroplasticity to improve defective function. NFB is an intervention which over time may result in long term changes of cortical activity [22]. BCI NFB can be categorised into the third category “promoting neuroplasticity to improve defective function”.

**Fig. 5:**
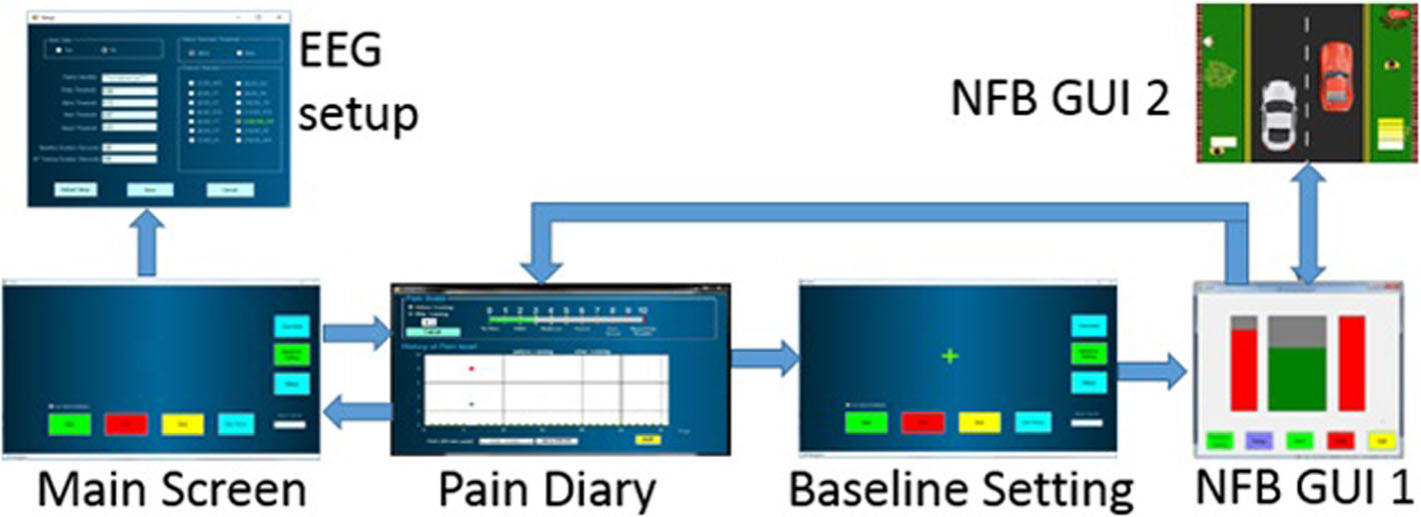
NFB application software. User access a pain diary screen from the main screen. Prior to NFB training participants enter their pain level and then go to the baselines setting screen. This is followed by NFB training using GUI1 or GUI2. After completing NFB training users return to the pain diary to enter the post NFB level of pain and return to the main screen to exit the application. The parameters in the EEG setup screen were typically set at the hospital by researchers and were password protected

Two different Graphical User Interfaces (GUIs) were available for NFB training (Fig. 5): The first GUI comprised of three bars, each presenting one frequency band which changed size and colour. A GUI with three bars was always used for the initial training, to establish a relationship between a mental strategy and EEG power in each frequency band [16]. The large middle bar represented the relative alpha power while side bars represented the relative power in theta and beta bands.

The second GUI modality was a car racing game. Participants had to increase the speed of a red car, relative to the speed of the white car. The speed of the red car was proportional to the combination of values of all three frequency bands and had three levels. The speed of the white car was proportional to the baseline EEG. The red car was fastest when all three frequency bands were regulated in the correct direction, i.e. when the alpha power was upregulated and beta and theta power were downregulated. The speed of the white car was constant.

The software was used in the following way (Fig. 5): After the optional setting of EEG parameters (selection of electrodes and frequency bands) participants filled in an electronic pain diary (pain level on VNS). Following this, they recorded their baseline EEG for 2 minutes. This was followed by NFB with the selected GUI. When training was finished, before exiting the software, participants had to enter their pain level in the electronic pain diary, a necessary step in order to exit the programme. Apart from raw EEG data from a channel selected for NFB and VNS values from the pain diary, the system also recorded information about time and frequency of use.

### BCI evaluation

#### Methods

Methods involved: (i) usability testing by end users and subsequent analysis of recorded data (EEG, electronic pain diary), (ii) inquiry methods including observation, interviews and questionnaires. A functional test of the NFB software application was performed prior to this study on able-bodied participants [45].

#### Measures

Measures of usability were divided into subjective (interviews, questionnaires, pain rating) and objective (EEG measurement). They were used to create qualitative and quantitative data. *Quantitative data* comprised of EEG recording, VNS pain rating via an electronic pain diary, questionnaires (Likert scale), and information extracted from the user activity log files (the frequency of use of the system and the number of daily sessions).

*Qualitative data* included questionnaires and semi-structured interviews.

Three validated questionnaires were used:

1. “Brief pain inventory” [56] was filled out at the beginning of the study to determine the location and the level of pain, independent of its origin.
2. “Neuropathic Pain Symptom Inventory” (NPSI) [57] was also administered at the beginning of the study to evaluate different symptoms of neuropathic pain. While this questionnaire is widely used for the assessment of CNP, it is not entirely adequate for patients with complete SCI injury as they might sometimes lack symptoms of allodynia and hyperalgesia, due to the absent sensation.
3. “Quebec User Evaluation of Satisfaction questionnaire” (QUESQ) [58] was used to evaluate the satisfaction with the system usage. QUESQ consists of twelve questions, eight related to the device and four related to services. Each question has 5 level of satisfaction (1-lowest and 5-highest). Participants were also asked to choose three out of twelve features that were most relevant for them.

Custom made questionnaires:

1. “Perceived Usefulness of a Device for Home-Based Treatment of Central Neuropathic Pain”. This was used to assess patients’ attitudes towards using new technology, testing perceived usefulness and the ease of use of the neurofeedback system. This questionnaire was based on a more general questionnaire: “Perceived usefulness, perceived ease of use, and user acceptance” [59].
2. “Attitude and Previous Experience with Non-Pharmacological Treatments of Neuropathic Pain”. This was used to assess patients’ attitudes and previous experience with other non-pharmacological treatments of CNP. Both questionnaires were administered at the beginning of the study.
3. “Neurofeedback System User Questionnaire” enquired about the participant’s experience of practicing NFB (perceived level of control, level of stress) and about specific technical issues of the EEG device which cannot be assessed by QUESQ. This questionnaire was administered at the end of the study.

The purpose of the non-validated questionnaires was to complement semi-structured interviews and to assure all participants were asked the same questions. It also allowed participants to answer the questions in their own time at home, due to the relatively limited time for semi-structured interviews.

Semi-structured interviews were organized with the participants during their check-up visit to the hospital or during the final assessment. The interviews covered the topics related to the experience of using hardware and software as well as the effect of the NFB on pain and other side effects and NFB strategies. Interviews were printed verbatim and analysed by two researchers independently (one of the researcher was not present when the interviews were taking place) to identify the main topics. Researchers then agreed on the main topics, presented in a two tiered mind map in the results section.

In BCI usability literature, it is common to present the measures according to the efficiency, effectiveness and satisfaction (user experience) (44). In the results section we will present all subjective and performance measures with respect to these three criteria.

*Statistical analysis* Mann Whitney U test was used to compare VNS pain intensity before and after neurofeedback and other demographic data. A significance level of *p* = 0.05 was adopted in all cases.

## Results

Out of twenty initially recruited participants, fifteen decided to take part in a home based NFB study (Fig. 2). All participants learned to self-manage BCI NFB and practiced NFB at home. Seven participants used the system for 2 months as required. Eight participants discontinued the study for the following reasons: unrelated health problems (*N* = 3), new caregiver (*N* = 1), moving home (N = 1), too long donning (> 15 min) (*N* = 2), broken device (*N* = 1). EEG data and pain diaries were collected from all fifteen participants. Nine participants (seven participants who completed 2 months NFB and two who had to discontinue the study early due to reasons unrelated to the study) took part in final interviews and filled out user experience questionnaires.

### Efficiency

*Efficiency* refers to the degree to which the product is enabling or hindering the task to be performed in a quick and economical manner. In this study, measures of efficiency were adopted such as the number of training and support sessions, BCI hardware and software donning time, percentage of EEG recorded in the home environment corrupted by noise and the number of NFB sessions per week.

Out of fifteen participants, only one participant required all four training sessions before taking BCI NFB home. Only two participants required additional training after taking the BCI NFB system home (Table 2). About half of the participants practiced NFB 1–3 times weekly while the other half practiced 3–5 times weekly (Table 2). A NFB session duration was 20–30 min, excluding donning and doffing.

**Table 2:** The Number of NF sessions that participants had with the g.USBamp and Epoch based BCI-NFB

Part. code	Nr g.USBamp sessions	Nr Epoch training sessions	Nr support sessios	Nr NFB home sessions /week	NFB home (weeks)
P1^a^	2	3	–	3–5	21
P2^ab^	2	2	–	1	8
P3	2	4	4	1–3	2
P4^ab^	2	3	–	> 5	12
P5^a^	2	3	–	> 5	14
P6	1	1	–	–	–
P7^a^	2	3	–	1–3	3
P8	3	–	–	–	–
P9	1	–	–	–	–
P10^a^	2	3	–	1–3	8
P11	3	2	–	1–3	4
P12	3	2	2	1–3	3
P13	1	1	–	–	–
P14	2	3	–	1–3	3
P15	2	2	–	–	–
P16^a^	2	3	–	3–5	8
P17^a^	2	3	–	3–5	9
P18^a^	3	3	–	3–5	3
P19	3	2	–	1–3	3
P20	1	3	–	1–3	2

^a^completed final assessment; ^b^ took part in Hassan et al. (2015) study

All fifteen participants demonstrated independent use of the system during a 2 weeks follow up session at the hospital. Two tetraplegic and four paraplegic participants required caregiver’s assistance.

During a follow up session, we observed BCI NFB hardware and software setup time for all fifteen participants. It took only two participants longer than 15 minutes to setup the system. The maximum self-reported setup time for both BCI NFB software and hardware (donning time) varied from 5 to 10 min (78%) to 10–15 min (22%), *N* = 9 participants.

On average 10–20% of EEG data had to be removed prior to analysis due to noise, demonstrating that participants managed to obtain reasonably good quality EEG signal during self-managed NFB. Analysis of EEG data was based on data from all fifteen participants.

Out of seven participants who used the system at their home for 8 weeks or longer, 5 could walk, one was sensory and motor complete paraplegic and one sensory and motor complete tetraplegic.

### Effectiveness

Effectiveness represents the accuracy and completeness with which specified users achieved specified goals in a particular environment. For BCI applications classification accuracy is a typical measure of effectiveness. Our BCI application did not have a classifier therefore for a measure of effectiveness we adopted (i) participant’s ability to selectively regulate specified frequency bands and (ii) the effect of NFB on pain. These two measures are related. Reduction in pain may be placebo effect if there is no accompanying self-regulation of brain activity. Successful NFB should modulate brain activity selectively, i.e. in selected frequency bands only. Nonselective upregulation or downregulation of whole frequency range might indicate changes in general arousal levels rather than engagement in a particular NFB protocol [60].

Although simultaneous control of all 3 frequency bands is a difficult task, participants were expected to at least upregulate (increase) the central frequency band (alpha), and to desirably downregulate the theta and beta bands. In a preceding study on able-bodied participants [45], we observed that people learned to upregulate the alpha rhythm sooner than to downregulate the theta and beta rhythm.

#### Ability to control neurofeedback

**Fig. 6:**
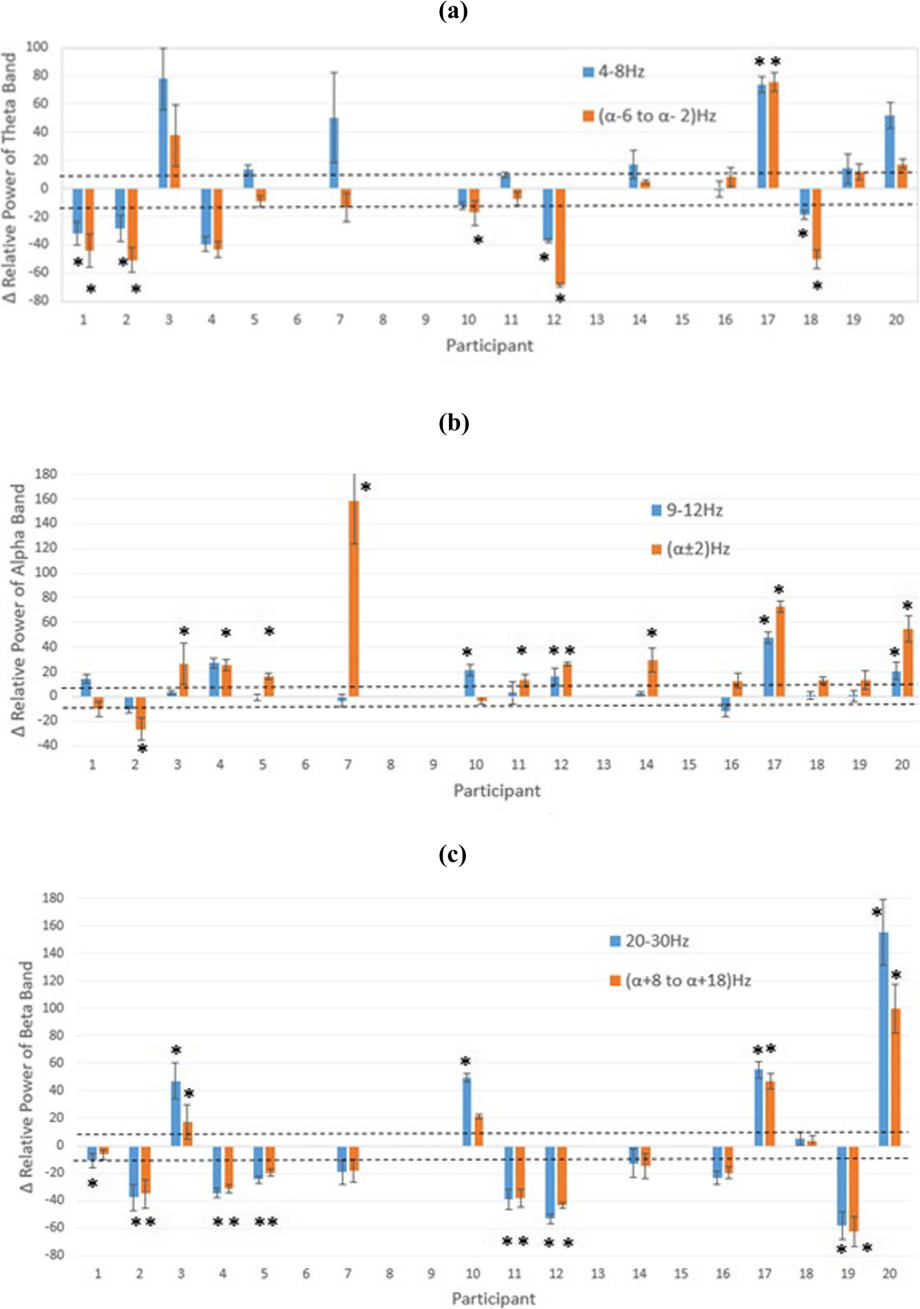
The average relative changes of PSD during neurofeedback over all NF training sessions (mean ± STD) for each single participant. The horizontal dot lines mark Δ10% change in relative power with respect to the baseline recording. Positive values show increase and negative values show decrease with respect to the baseline power. Note that the NFB task was to increase the power of alpha for 10% or more and to decrease the power of theta and beta band for 10% or more. **a** Theta (4–8 Hz) in blue, and “individual” theta in orange. **b** alpha (9–12 Hz) in blue and individual alpha in orange colour. **c** higher beta (20–30 Hz) in blue and “individual” higher beta in orange. The results of participants 6, 8, 9, 13, and 15 are missing because they did not use the BCI NFB at home. Asterisks show statistically significant differences with respect to the baseline (*p* = 0.05)

Figure 6 shows the average percentage change (mean ± std) for each participant in theta, alpha and higher beta frequency bands during NFB as compare to the baseline. Positive values mean that the power in selected band increased while negative values mean that the power decreased during NFB. Power was calculated in two ways: in fixed frequency bands as provided during NFB, theta (4–8 Hz), alpha (9-12 Hz), higher beta (20–30 Hz) and with respect to the individual alpha peak α_p_ as alpha band (α_p_-2 Hz, α_p_ + 2 Hz), theta band (α_p_-6 Hz, α_p_-2 Hz) and higher beta band (α_p_ + 8 Hz, α_p_ + 18 Hz). A dashed line represents 10% change during NBF, which was the main training outcome. In addition, statistical analysis was performed over all training sessions, to assess whether NFB consistently modulated EEG power in a desired direction. This was a somewhat conservative approach as it also included early sessions while participants were still learning the NFB technique.

Figure 6 shows that participant’s performance, particularly in the alpha band, was more successful when power was calculated with respect to the individual alpha band (α_p_). Nine out of fifteen participants significantly increased their individual alpha band compared to four who increased the 9–12 Hz band. If two participants with previous experience in NFB are excluded from the analysis, eight and four participants significantly increased alpha band power in the individual and fixed bands respectively.

The choice of individual bands had less impact on the theta and beta frequency range. Five participants significantly downregulated their individual theta band (α_p_ − 2 to α _p_ − 6 Hz) while four significantly downregulated fixed theta band (4–8 Hz). Five participants significantly downregulated individual higher beta band (α_p_ + 8 to α _p_ + 18 Hz) while four significantly downregulated fixed higher beta (20–30 Hz) band. However, four participants consistently modulated beta band power in the wrong direction, indicating that the beta band was hardest to regulate. If case that two participants with previous experience with NFB are excluded from the analysis, four and three participants significantly reduced their theta band power in the individual and fixed bands respectively while four and five participants significantly reduced their higher beta band power in the individual and fixed bands respectively. One of these two participants non -selectively increased power in all frequency bands while the other followed the rules and increased the alpha and decreased the theta and beta band power.

Fourteen out of the fifteen participants significantly modulated at least one frequency band, eight significantly regulated at least two bands in the desired direction and only one significantly regulated all three bands in the desired direction. Three participants non-selectively increased or decreased EEG power over the whole spectrum.

When only changes in the mean value of the EEG power were observed, fourteen out of fifteen participants were able to increase by more than 10% either their individual α or 9–12 Hz alpha band power. Seven participants decreased their theta band power (individual or 4–8 Hz) by 10% or more and ten participants decreased their beta band power. This indicates that alpha band power was the most successfully regulated frequency band.

**Fig. 7:**
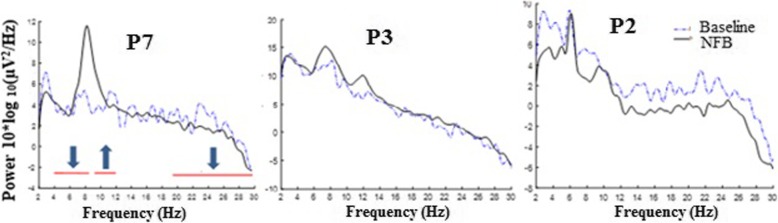
Power spectrum density during baseline (PreNFB, dashed line) and during NFB (solid line) over one session in three representative participants

Figure 7 shows several different scenarios during one representative NFB session. Participant P7 successfully upregulated the individual dominant frequency while downregulating theta and beta band power. Participant P2 downregulated the individual theta and beta band, his individual dominant peak at 6 Hz remained unchanged but a new peak around 10 Hz emerged when the 6–8 Hz power was reduced. Finally, participant P3 further increased the existing dominant peak at 6 Hz while also creating another peak at 10 Hz.

#### The effect of NFB on pain intensity

Twelve out of fifteen participants achieved a statistically significant reduction in pain (Mann Whitney U test, *p* = 0.05). This reduction was clinically significant (> 30%) in eight participants [61]. Out of ten participants who significantly upregulated the alpha power (α_p_ or 9–12 Hz), eight had significantly reduced pain. Out of the four remaining participants who reported a statistically significant reduction of pain, two significantly downregulated both the theta and beta bands. Out of eight participants who achieved clinically significant reduction of pain, five were able to walk, two were paraplegic and one tetraplegic wheelchair users.

Taking into account all 20 participants recruited in the study this gives an efficacy of 40% (8 out of 20). When excluding the two participants who had previous experience with NFB, the efficacy is 39% (7 out of 18), being very similar to the efficacy of the whole cohort.

There was no significant correlation between the level of pain and the level of injury (*p* = 0.6949, r = 0.0935), confirming results of previous studies [2]. The level of pain was not significantly correlated with a time since injury though the *p* value was close to the significance level (*p* = 0.0631, *r* = 0.4231) indicating that pain might get worse over time. No significant correlation was found between the level of pain and the reduction of pain on the VNS (*p* = 0.81, *r* = 0.65), the initial level of pain and the dominant alpha frequency (*p* = 0.4522, *r* = − 0.2101), the dominant alpha frequency and the reduction in pain during NFB (*p* = 0.9703, *r* = − 0.0105) and time since injury and the reduction in pain during NFB (*p* = 0.9701, *r* = 0.1010). Likewise, there was no significant difference in the initial level of pain between walkers (ASIA D) and non-walkers (ASIA A and B) (Wilcoxon *p* = 0.7528), nor between participants with incomplete (ASIA B and D) and complete (ASIA A) injury (Wilcoxon *p* = 0.6242), though a previous study showed that people with ASIA A-complete injury have more severe pain than those with the incomplete injury [48] Table 3.

**Table 3:** Pain intensity before and after NF: median, (quartile 1, quartile 3). The statistically significant level *p* = 0.05 (Mann Whitney U test)

No.	Pain Intensity		Change in Pain Intensity (%)	*p*-value
	PreNFB (VNS) mean ± STD (median)	PostNFB (VNS) mean ± STD (median)		
P1^a^	8.7 ± 1.2 (9)	6.0 ± 1.0 (6)	−31%^c^	0.007
P2^ab^	8.5 ± 0.7 (8)	5.5 ± 0.7 (5.5)	−35%^c^	0.001
P3	8.7 ± 0.6 (9)	5.3 ± 0.6 (5.5)	− 38%^c^	0.002
P4^ab^	3.0 ± 0.5 (3)	2.4 ± 0.5 (2.5)	−18%	0.5e^−5^
P5^a^	5.1 ± 0.6 (5)	4.5 ± 0.6 (4.5)	−10%	0.5e^−6^
P6	–	–	–	–
P7^a^	4.5 ± 0.9 (4.5)	3.5 ± 0.9 (3.5)	−22%	0.001
P8	–	–	–	–
P9	–	–	–	–
P10^a^	9.0 ± 0.7 (8)	5.5 ± 1.8 (5.5)	−38%^c^	0.04
P11	4.0 ± 1.0 (4)	3.3 ± 0.6 (3)	−15%	0.08
P12	7.25 ± 0.5 (7)	4.3 ± 0.5 (4)	−34%^c^	0.002
P13	–	–	–	–
P14	5.3 ± 1.0 (5)	3.7 ± 0.8 (4)	−28%	0.003
P15	–	–	–	–
P16^a^	2.8 ± 0.5 (3)	0.7 ± 0.6 (1)	75%^c^	0.5e^−5^
P17^a^	5.4 ± 1.0 (6)	2.9 ± 1.3 (3)	−46%^c^	0.5e^−11^
P18^a^	5.3 ± 2.1 (5)	4.7 ± 2.3 (4)	−15%	0.09
P19	5.8 ± 1.7 (5)	2.3 ± 1.7 (2.5)	−65%^c^	0.0006
P20	7.3 ± 0.6 (7.5)	6.7 ± 0.6 (6.5)	−9%	0.09

*VNS* Visual Numerical Scale (VNS, 0 = no pain, 10 = worst pain imaginable), *PreNFB* before daily NFB session, *PostNFB* after daily NFB session.; ^a^ completed final assessment; ^b^ took part in [21]; ^c^ clinically significant reduction of pain, larger than 30%

As part of the final assessment, participants (*N* = 9) were asked how long they experienced a reduction in pain following NFB training, Their answers were: never *N* = 1, during NFB only *N* = 1, for at least 1 h following NFB *N* = 1, for remainder of the day *N* = 1, day and night *N* = 2 and longer than 1 day *N* = 2. Note that these were not the same participants as those who reported the maximum reduction in pain.

*Transfer learning*: learning NFB techniques without a device is one of the important goals of NFB [16]. In our previous study we recorded EEG in participants experienced with NFB while using a NFB strategy without the device. They regulated their brain activity in a very similar manner to actual NFB. Although in this study we did not explore transfer learning systematically, three participants reported positive effects of visualising NFB on pain and on related spasm.

One participant said “*I wear headphones at work and the sensation of wearing the headphones is similar to the headset. If I’m at work and the spasms kick in, I just visualise the screen and within 5–10 min the spasms have gone.”*

It is however important to notice that it is necessary to practice NFB with the device at least once a week to keep ths ability.

### Usability inspection

Usability Inspection comprises two elements, Performance Measure and User Experience, i.e. Satisfaction [46]*.*

#### User experience

At the beginning of the study, upon demonstrating a wearable BCI-NFB for the first time to participants (*N* = 15), they were asked to rate perceived usefulness and ease of use of the device as well as their general attitude towards using novel technology. Participants who had a caregiver filled in the questionnaire together. Results show that participants are “early adopters” of technology [62]. While high average scores might increase the risk of bias, for this type of study which requires relatively high commitment, it was necessary to have highly motivated participants.

**Fig. 8:**
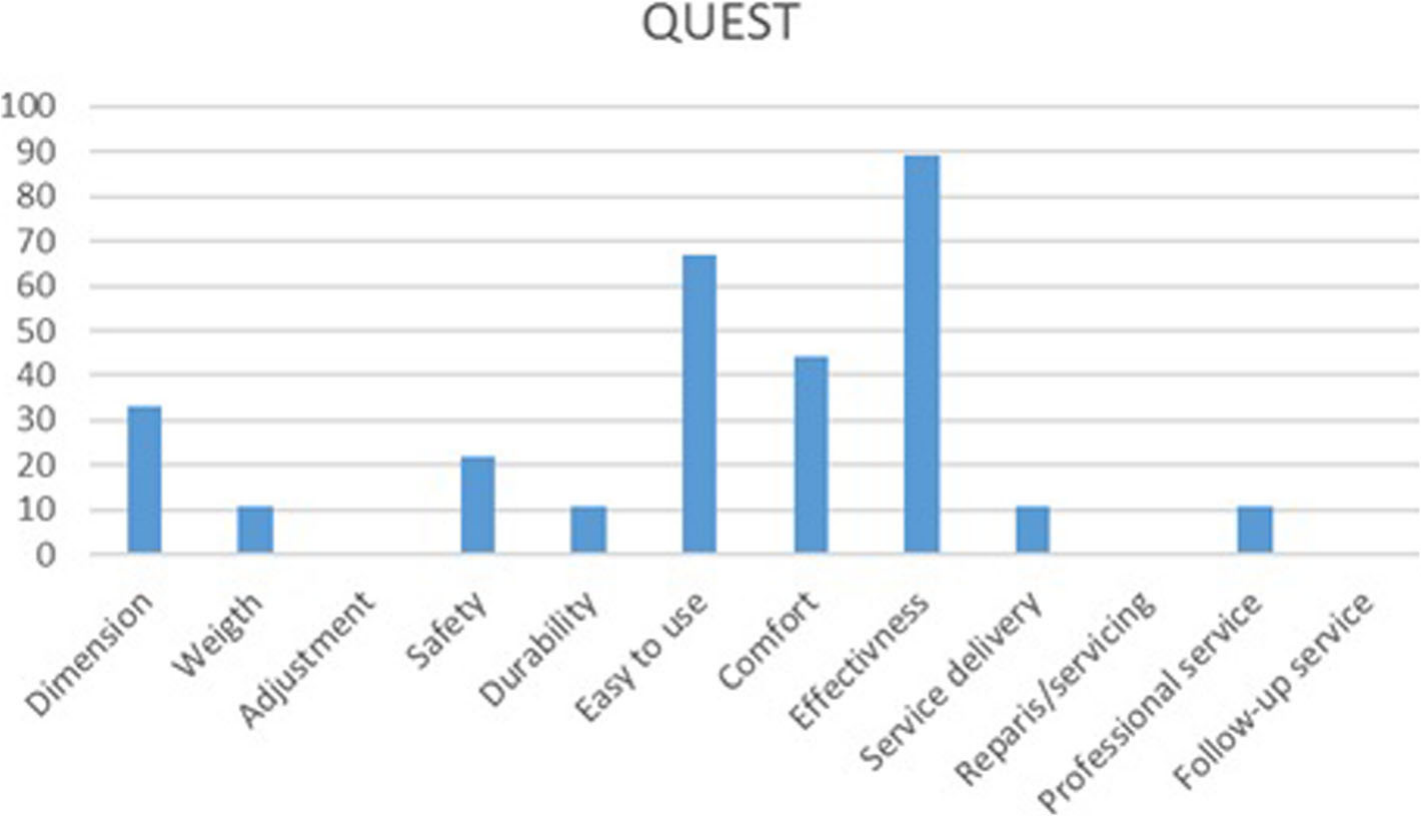
QUEST User priorities, in percentage. Number of participants *N* = 9

QUESQ questionnaire (*N* = 9) was filled out on the final assessment. This is a 5 point questionnaire with 1 being the worst and 5 the best mark. The results of questionnaire showed that participants were on average satisfied with the device 4.5 ± 0.8 (median 4.6) and with the service provided 4.9 ± 0.3 (median 5). The cumulative score for all questions was 4.6 ± 0.5 (median 4.6). Their main priorities were: effectiveness, ease of use and comfort (Fig. 8) (Table 4).

**Table 4:** Perceived usefulness and the ease of use of BCI (Q1-Q4) and attitudes towards using novel technology, Q5-Q7

Question	Range	Mean ± STD (median)
Q1. In your opinion, how easy is it to understand the main purpose of the EEG-tablet system?	1 very easy 10 very hard	2.3 ± 1.4 (2)
Q2. Please rate how much you agree with the statement: I would like to have this device but I am not sure if my caregiver and I would understand how to use it	1 very false 10 very true	1.6 ± 1.4 (1)
Q3. Please rate how much you feel convinced that the device might help reducing your pain.	1 not at all 10 very much	8.9 ± 1.3 (9.5)
Q4. How easy do you feel that it is to use this device on a daily basis?	1 very hard 10 very easy	7.8 ± 2.1 (8)
Q5. Please rate how you would feel if other people saw you wearing the device at home	1embarrassed 10 amused	8.9 ± 1.3 (9.5)
Q6. Please rate how you would feel if other people knew that you were using the device at home	1 embarrassed 10 amused	8.5 ± 1.9 (8)
Q7. Please rate your attitude towards using novel technology (e.g. computers, phones, other gadgets)	1 avoidance 10 excitement	7.7 ± 1.5 (8)

To assess the “burden of treatment” participants (*N* = 9) were asked how much the NFB interfered with their daily routine and which factors influenced the frequency of use of BCI NFB. Practicing NFB did not interfere at all (67.5%) or somewhat interfered (37.5%) with their daily routine. The single most important factor influencing frequency of use was “other health conditions” (*N* = 9), followed by “available time” (*N* = 6), “pain intensity (*N*=5), “caregiver time” (*N* = 3) and “mood” (*N* = 3). No other factors were mentioned. During the study, twelve out of the fifteen participants reported at some point to the research team unrelated health problems which to a greater or lesser extent influenced the frequency of use of BCI NFB.

Participants on average reported that they were most of the time in control of NFB (7.7 ± 2.3, 1 = never, 10 = always, *N* = 9). On average participants felt mildly stressed when doing NFB for the first time in their own at homes (2.4 on a scale 1–10, 1 being minimum stress).

It should be noted that 6 participants did not provide responses, so these high scores might be biased (Table 5).

**Table 5:** NFB user experience Q1, Q2 presented as mean ± STD (median), Q3, Q4 presented as percentage

Question	Range	Rating
Q1. How often did you feel that you were in control of neurofeedback?	1 never	7.7 ± 2.3(8)
	10 always	
Q2. How stressful was it the first time you had to use the device in your own at home?	1 not stressful at all 10 extremely stressful	2.4 ± 2.5 (1)
Q3. Did the pain treatment interfere with your daily routine?	a) interfered a lot, b) interfered, c) sometimes interfered, d) Did not interfere at all	- - 37.5% 62.5%
Q4. How long on average did it take you/your caregiver to setup the whole system?	a) 5–10 min, b)10–15 min c) 15-30 min d) > 30 min	78% 22%
Q5. What factors influenced how often you used the device? (circle as much as appropriate)	a) Intensity of pain, b) Free time, c) Mood, d) Available time of my caregiver,	5 6 3 3
	e) Other health problems f) Other (explain).	9 0

### Interviews

**Fig. 9:**
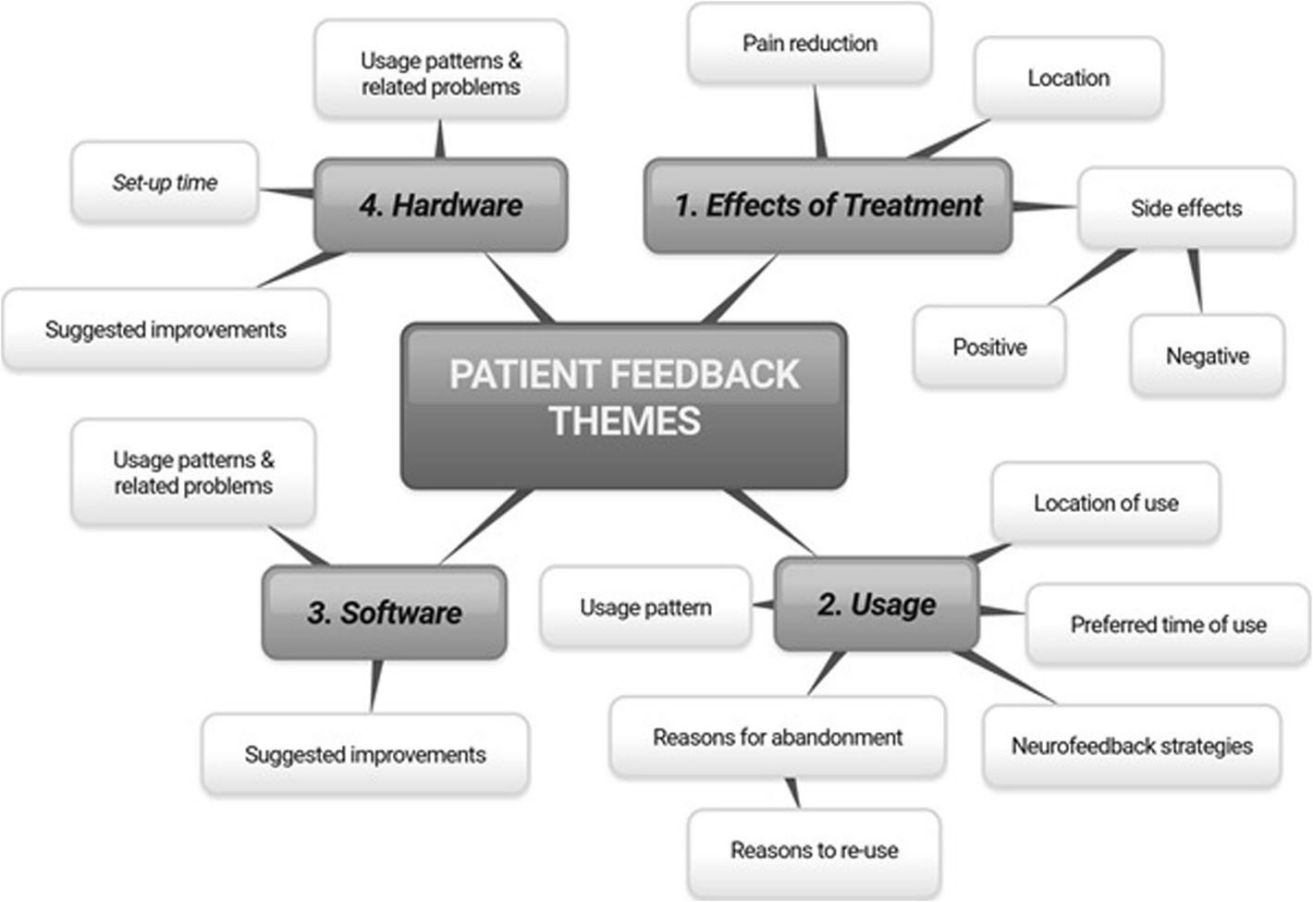
Main themes from interviews with participants

Interviews were organised at the first check-up visit about 2 weeks after starting NFB (*N* = 15) as well as during the final assessment (*N* = 9). We also used information from emails and SMS messages in this analysis. Four main themes were identified: effects of treatment, usage, hardware and software. Within these topics further subtopics were identified. Figure 9 shows two classification tiers.

Theme “Effects of treatment” had three categories:

- Reduction of pain
- Location of perceived pain (at or below level of injury: torso, arms, legs, hands, feet), pain reduction, pain descriptors (burning, squeezing, stinging, shooting). This is how one of the participants described the effect of NFB “*This training is longer than I would do my relaxation and it has an immediate effect in terms of lowering pain. What is new for me is that there is some residual effect that lasts three to four days following training where the pain level is lower and different to what I would normally experience”.* The other participant reported experiencing the greatest reduction in pain several hours after treatment *“You don’t notice the pain getting less until maybe an hour later, … , then an hour later the pain gets less and as the day goes on and night goes on pain gets less and less”*
- Side effects of NFB: negative - hypersensitivity in the feet, occasional headaches; positive - better sleep, less spasm, improved foot sensation, pleasant warmth replacing a burning sensation. The same participant who reported visualising NFB when wearing headphones at work said “*Spasms at work have diminished greatly. And I mean greatly. And I’ve only just clicked that it’s since I’ve started using this [BCI-NFB] that when I put the headphones on at work, the spasms diminish*”. Another participant wrote in an email “*I am still getting a brilliant sleep with no spasms even to the point I have slept in a few mornings.”*

Theme “Usage” had five categories:

- Preferred time of use (morning, evening, when pain is worst). Most participants practiced NFB in the evening because that was when they had free time and also when pain was worst. Reducing pain in the evening improved sleep.
- Location of use (bedroom, any place with no distractions)
- Usage pattern (20–30 min daily, 1–7 times a week). The usage pattern depends on the available time of a caregiver (professional caregivers were available for several hours a day and their priority was to provide assistance with activities of daily living; family caregivers were more flexible as they were typically not helping with personal hygiene and had more time). One person with tetraplegia changed caregivers during the study and the new caregiver was trained to use BCI-NFB.
- Reasons for abandonment: unrelated health issues e.g. infections, changes in daily routine such as moving home, holidays and negative opinion of a family doctor.
- Usage strategy: in addition to relaxation which is recommended for NFB in general, participants often mentioned “thinking of happy memories” e.g., favourite holiday on a beach, riding a horse etc.

Theme “Software” comprised of two categories:

- Usage pattern and related problems: forgetting instructions, small font warning messages. Some participants also reported that the system was easy to use. The preferred GUI for all participants was the one with bars.
- Suggested improvements: step-by-step instruction on screen, better measure of daily performance, increased font size of warning messages

Theme “Hardware” comprised of three categories:

- Usage patterns and related problems: no problems, awkward to put on, slipping from the head, uncertain about the quality of EEG, hard to get low impedance, robustness, headset breaking, caregiver availability vs NFB training time
- Setup time: ranging from 5 to 30 min (two participants who reported 30 min withdraw early)
- Suggested improvements: dedicated headset for pain treatment (to be placed over the central cortex), increased robustness, unambiguous location on the head.

#### Performance measure

**Fig. 10:**
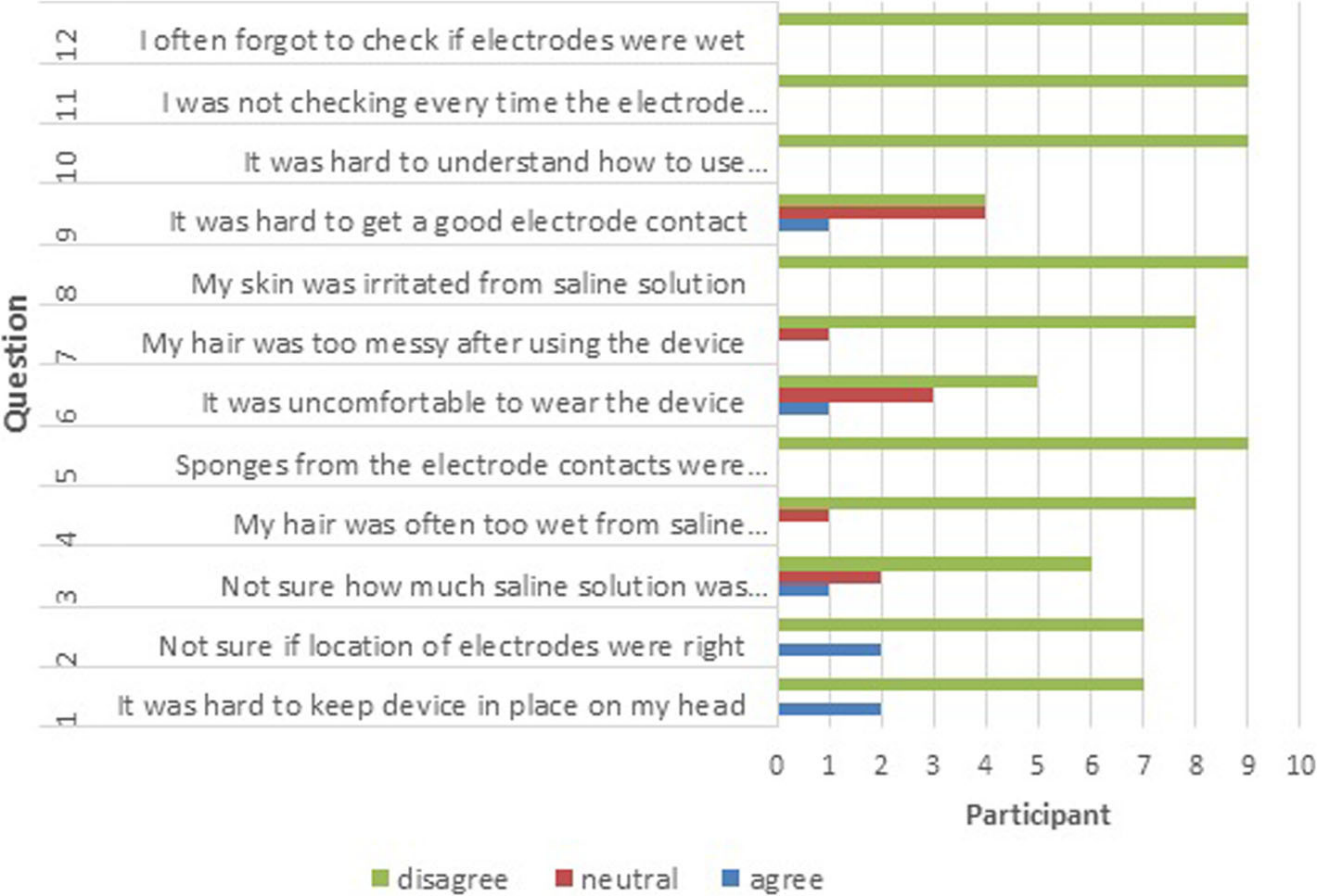
Experience with using BCI hardware (*N* = 9)

This has to some extent already been addressed under interview topics Software and Hardware. In addition, nine participants provided feedback about technical usability of the EEG headset, as a part of the final assessment (Fig. 10). The answers indicate that the main technical problem was to determine the correct location of electrodes (2/9) and to prevent the device from slipping from the head (2/9). Issues most often reported in the interview during the check-up sessions were: “How to achieve better contact with electrodes/low impedance”, “How to start the device?” (forgetting to switch on a headset, forgetting to use a dongle), “How to leave the software?” (forgetting to fill out a mandatory electronic pain diary). Two devices got damaged during home use but in only one case the damage prevented further use. However, the results show that participants were overall satisfied with the hardware. Again, it should be noted that six participants have not answered these questions and that these participants used the system for less than 2 months.

## Discussion

In this study we investigated whether people with SCI and with CNP can practice NFB on their own or with the help of their caregivers at home. Although all participants suffered from long standing CNP their level and completeness of injury varied, which enabled us to investigate the needs of people with different levels of disability. Data had been collected in a home environment, experiencing environmental noise, while using a consumer grade EEG.

BCI user centred design has four stages [44]: understand and specify the context of use, specify the user requirements, produce design solution to meet user requirements and evaluate design against requirements. Most usability studies present only the last stage.

We have adopted a usability framework which was developed based on previously published studies [46]. This study demonstrates that it is also suitable for presenting the original data. We made only minor modifications to include participants in a wider sense, including the research team and their experience with training and technical support throughout the study. While technical support is an integral part of QUEST is it typically not included in BCI usability studies. Although this study only touches on that topic, we suggest that in the future service support should be an integral part of home based usability studies.

The challenge in developing home based BCI was to create a system that was easy to use, reliable and accessible to people with disabilities and their caregivers. Notwithstanding was a requirement to create an inexpensive system by using consumer grade EEG and a freeware software platform, in order to provide at the same time BCI to a relatively large number of end users within a limited budget. We used C++ rather than Matlab which is cheaper and adequate for tablet computers. In the future C++ can be used with mobile phone applications.

The number of participants in the study (20) was relatively small, compared to studies focused solely on the effectiveness of a therapy. On the other hand, twenty participants is a relatively large number for BCI usability studies on patients, which often had less than 10 participants [46, 63, 64]. While we demonstrated the effectiveness of NFB for a relatively niche group of patients, other aspect of usability, such as efficiency and user satisfaction are of relevance for a wider patient community that might use BCI for home based treatments. In addition, under a hypothesis that thalamo-cortical dysrhythmia is the core of CNP [65], and that it has EEG markers which are independent of the aetiology of the neuropathic pain [66] one might argue that similar NFB protocol might be effective in other patient groups.

Apart from Daly et al. [34] study, our study is one of the rare usability studies of a therapeutic use of BCI. Our participants used BCI on their own for 2 months, that provided the opportunity to observe not only experiences of patients, but also of the research team providing service support (communication channels, frequency of communication, technical issues). During the study, the main challenge for the research team was to provide sustained technical support throughout the study and to constantly keep in touch with a relatively large number of people without having a dedicated person solely for this task. Continuous communication and encouragement was essential because small changes in daily routine were often reasons for temporarily stopping treatment. Larger yet unavoidable obstacles were frequent health problems which are common in people with SCI.

### Effectiveness

Five out of twenty initially recruited participants were either not able to control NFB or found the protocol too demanding (difficult to use or time consuming). However, all fifteen interested participants learned how to use the system within four sessions. There are several factors which contributed to successful learning. We created custom made user manuals and instructed participants to take photos or videos of themselves as a reminder of the setup procedure. Other research groups have also reported recording EEG from the central cortex using Emotiv, however this was performed by researchers rather than by participants [63]. An additional facilitating factor was that the consumer EEG device used has been designed for non-professionals and has additional sources of information on the Internet. The donning time in this study was up to 15 minutes, comparable to results of studies managed by caregivers only [31]. This time would probably be reduced if the headset had been originally designed to cover the central cortex. During NFB with the Epoch, we recorded EEG from one electrode location only to minimise the setup time, though we instructed participants to check impedance levels of all electrodes.

Participants reported low levels of stress when using BCI for the first time at home on their own. We did not use NASA task load index [67] because asking participants to fill this questionnaire regularly at home would likely result in a low level of compliance. The answer that we collected was based on recollection, during participants’ check-up visits to the hospital.

Although previous studies showed that Epoch had reasonable performances [63, 68], they were performed under laboratory conditions. Our results showed that in the home environment, only 10–20% of recorded data was very noisy, which is an extremely encouraging result for future real world BCI applications. On the other hand, the physical design of the headset which was not originally created to record EEG from the central cortex was the major problem, in particular for those with smaller heads or thick hair. To the best of our knowledge, at the moment there is no other consumer grade multichannel EEG device that is designed to record brain activity with non-gel electrodes from the sensory-motor cortex, costing under £1000. Inexpensive technical solutions for EEG recordings (e.g OpenBCI) could be used with custom made headsets but do not provide easy solutions for non-professional users. As noted by Miralles et al. [30] the price of BCI is currently the biggest restricting factor for large scale usability studies in the home environment.

Most participants preferred training with bars rather than with cars. One reason might be that the car game was not as entertaining as games available with commercial NFB devices but the other reason could be that the GUI with bars provided clearer response-reinforcement association which could be overshadowed by more complex games [16]. In this study a threshold for NFB was fixed throughout the training, based on the baseline measurement for that day. NFB practitioners occasionally use a “moving” threshold based on the most recent performance in order to provide a reward, irrespective of patient performance. This may however lead to training in an undesired direction [16] and would make later quantitative analysis difficult.

### Efficacy

Efficiency was measured by the ability to control NFB and to achieve reduction in pain. The principle of NFB is operant conditioning, a learning strategy that increases a preferred behaviour and decreases the undesired behaviour by providing a reward or punishment [69]. Results from the literature show that similar to the general BCI illiteracy problem, some people cannot learn to use NFB [70]. Prior to this study, we tested the NFB protocol with Emotiv in one 30 min session on eighteen able bodied people [45]. Fourteen participants increased the alpha power by more than 10%, eight decreased theta and seven decreased higher beta by more than 10%. In the current study, only two participants were not able to control NFB.

An important measure of successful NFB is selectivity, i.e. the ability to regulate only selected frequency bands rather than to increase or decrease the whole frequency spectrum [60]. Twelve out of fifteen participants selectively modulated frequency bands as required by NFB while three increased or decreased the whole frequency spectrum. Similar to able-bodied participants, participants with CNP were most successful upregulating the alpha band with twelve out of fifteen participants upregulating it by more than 10%, seven downregulated theta and ten downregulated their beta band power by more than 10%.

An important observation is that most participants actually upregulated their individual alpha range (6–8 Hz) which on average was lower than in able bodied people, while some participants created two ‘peaks‘, one around their individual alpha and the other in the 9-12 Hz range. A reduced dominant alpha frequency was reported in several previous studies [8–11] and has been attributed to the thalamo-cortical dysrhythmia [68]. In our recent study [12] we found that the reduced alpha power and reduced dominant alpha frequency are both markers of “future” CNP. This NFB protocol was aiming to increase not only the alpha power but also the dominant alpha frequency. However, results of this study indicate that alpha amplitude rather than alpha frequency is related to the reduction in pain. We did not notice an issue with individual alpha bands when previously testing the NFB protocol on able-bodied [45] because their average dominant alpha frequency was within 8–12 Hz.

The efficacy of NFB was 40% i.e. in 8 out of 20 initially recruited participants, the reduction in pain was clinically significant, i.e. larger than 30% [61]. These are encouraging results, that should be used to estimate the effect size in a future larger randomised clinical trial. Although results of a single trial cannot be directly compared to the results of a meta-analysis, the meta-analysis results of other pharmacological and non-pharmacological treatments should serve as a desirable target. According to a Cochrane database study, gabapentin, a widely used pharmacological treatment of CNP [71], has an efficacy of 50%. The efficiacy of NFB should also be compared to the other neuromodulatory treatments of CNP such as rTMS and tDCS, although rTMS is still not available for home use. A recent Cohrane review showed that rTMS on average results in 12%, while tDCS results in 17% short term relief in pain [72]. That study adopted 15% as a clinically relevant reduction in pain, meaning that only tDCS may result in a clinically significant reduction in pain. If we addopted 15% as clinically significant reduction of pain in this study, then 13 out of 20 participants (65%), would achieve clinically significant reduction of pain.

Looking at the relationship between NFB and the reduction in pain, eight out of twelve participants whose pain was significantly reduced also had a significant increase in the alpha band power. Out of the remaining four patients who had a significant reduction in pain without significantly upregulating the alpha power, two significantly downregulated both theta and beta band power. A multivariate analysis, which was outwit the scope of this usability study may reveal more complex relations between different NFB parameters and the reduction in pain.

For six out of fifteen participants who could walk (ASIA D) pain was the major cause of restricted activity. Five of them used the system for 2 months and achieved statistically significant reduction of pain. This indicates that being able to self-administer NFB when and where needed might have a positive effect on the compliance and the effectiveness of NFB.

A limitation of this study is that there was no explicit test for placebo effect. There are two options to test for placebo: the first one is to occasionally blindfold participants to practice NFB with a pre-recorded session or from another area of the cortex. We tested both approaches in our previous laboratory based study, but this would have been hard to achieve in the present study because of infrequent direct contact with patients and because of transfer learning. The other option to test for placebo effect is to have a control group, which might be unethical in long-term studies due to the level of commitment expected by participants.

The presence of a trusted authority (a researcher or a therapist) may also have a placebo effect [16]. In this study however, participants practiced NFB on their own. We cannot however exclude that shifting locus of control from external to internal did not have a placebo effect. On the other hand one can argue that sham neurofeedback provided from an active electrode might also induce a similar placebo effect.

In this study, instead of placebo test, we collected patient self-reported descriptions of sensations related to NFB (tingling, pleasant warm sensation etc) [21] that accompany reduction in pain, to quickly assess whether there was any effect of NFB. We showed that out of 10 participants who significantly upregulated the alpha power, eight achieved significant reduction of pain. We also checked whether pain reduction lasted beyond the NFB training.

In this study NFB training was provided from C4 which is located over the primary motor cortex of the left hand. The same location that proved to be most effective for rTMS and tDCS treatments of CNP [15]. It is believed that the mechanism of action of these treatments is through collateral neural branching [73] which explains why it is not necessary to apply NFB for CNP in a somatotopic manner. In [21] we showed that NFB from C4 also affects the motor cortex symmetrically over the contralateral side (electrode location C3).

*Satisfaction* is defined by the degree a product is giving contentment or making the user satisfied. Judging by the results of questionnaires assessing participant’s attitude towards novel technology and the perceived usefulness of BCI-FES all participants were “early adopters” of technology. These questionnaires were answered jointly by the participants with SCI and their caregivers and they jointly provided high scores. It shows that for adoption of new assistive or therapeutic technology it is important to motivate both patients and caregivers. An important issue emerged during interviews, that caregivers are typically not available for the whole day and that quick BCI donning is extremely important because of the overall limited time that caregivers could devote to the therapy. Previous studies investigating BCI priorities in people with SCI included only questionnaires without actual use of BCI [74]. For this reason this study might provide useful information regarding the design of home based BCI studies with the SCI population, beyond NFB.

Results of QEUST showed that participants who completed the study were on average very satisfied with both the device and technical support (services) provided by the research team. According to QUEST the main priorities were functionality, ease of use, comfort and dimensions, which are similar to user priorities from other BCI usability studies. A very encouraging result is that most participants did not feel stigmatised by using a headset in front of other people at home. It should be however noted that not all participants completed the questionnaire and that three participants initially withdrew from the study as they perceived the BCI NFB system to be too complex. Fifteen out of twenty initially recruited participants, who stayed on the study were “early adopters” of technology and their level of commitment and satisfaction might not necessarily translate to more general users of this technology. However, feedback from “early adopters” is valuable for improving the technology to make it acceptable for more general patient population.

We identified four main themes in the semi-structured interviews: effect of NFB, usage pattern, problems with hardware and problems with software. The main effect of NFB was a reduction in pain but participants also reported other positive side effects such as reduced spasm and reduced foot drop, improved sensation and proprioception. Finnerup [75] recently suggested similarities in neuronal origin of spasticity and CNP, such as differentiation of supraspinal neurons. Thus, a neuromodulatory intervention applied to the motor cortex, which affects one might affect the other phenomena. Tingling and a pleasant warm sensation were also reported frequently accompanying NFB, and these were also typically the first symptoms to NFB. This is indirect evidences of modulation of the sensory-motor cortex that is typically overactive in people with SCI and SCP [6]. Similar side-effects were also reported in our previous study [21]. Side effect reported in this study should be interpreted with caution, because they were not systematically monitored. Future, large scale, trials should incorporate sensory and spasticity test in the protocol. Alternatively, a NFB study that focuses solely on spasticity in people with SCI with preserved mobility would provide results that are more conclusive.

We did not separately assess satisfaction of people with SCI and their caregivers because only six out of fifteen participants had a caregiver and because caregivers’ roles varied from patient to patient. Only two tetraplegic patients required a caregiver to initiate NFB software.

An interesting observation was that patients often considered happy episodic memories during NFB, as a mind wandering strategy, indicating the involvement of the default mode network [76]. This was unfortunately not possible to explore further due to single channel EEG recording.

The single main factor affecting the frequency of use of BCI was other health related issues which are fairly common in people with SCI (e.g. urinary tract infection, skin problems etc.). Our ethical permission did not allow us to get insight into all medications that participants took. However we have checked that during study they did not change dosage of pain medications, did not start any non- pharmacological treatment and did not aquire any neurological issues, that would affect their EEG. We also noticed that any disruption to the daily routine could potentially disrupt the usage pattern, indicating the need for regular monitoring in the early phase of adopting new technology. Other factors influencing frequency of use were available time and pain intensity. However, in some cases family doctors (general practitioners) were concerned about possible side effects of BCI as they were unfamiliar with it. This indicates the importance of educating healthcare professionals i.e. trusted persons for a wider user acceptance of BCI technology.

Problems with hardware mainly arose because the EEG headset had to be tilted, i.e. it was not placed on the head as it was designed for. Some previous studies also used this setup to record EEG from the sensory-motor cortex [63]. Patients frequently used elastic bands to keep the hardware in place. Getting a good electrode contact with wet electrodes was also an issue mostly during the first few sessions but was a reason for abandoning the study by patients with thick or long hair. Forgetting to use a dongle or switch the device on were also frequent reasons for seeking help from the research group. Online monitoring of signal quality and automatic noise removal would be a bonus for any future BCI hardware designed for home use [77].

Software was not a source of concern because it has been thoroughly tested on able-bodied volunteers [45]. The largest problem during the first few sessions was remembering the instructions. Based on participants’ feedback, we plan to incorporate the following features in the next version of the NFB software: simpler measure of NFB performance with an electronic diary (current version does not have a diary); step-by-step instruction which could be switched off once participants become experienced with the software, larger font warning messages, a simplified one page software instruction and a car game GUI with better graphics. The NFB software has been designed in a way that it is independent of the hardware so in the future it could be used with custom-made headsets which are designed to cover the sensory-motor cortex.

Another feature that would be useful to incorporate is an audio warning related to the on-line monitoring of the signal quality. Interestingly, most participants reported that they could recognise from the dynamic of the visual feedback with bars whether they were successfully doing NFB or if the signal was of poor quality. That might explain why GUI with bars was preferred by all users, as it provided direct control of all features that should be controlled by NFB. We did not specifically test for the locus of control [78], though “being in control of pain” was probably a major drivers behind this treatment.

## Conclusions

The study demonstrates the feasibility of home-based patient and caregiver managed NFB therapy for CNP. The results of this study should encourage other researchers to take BCI from labs and hospitals to patients’ homes and should inform the developers of wearable consumer BCI devices. The study also demonstrates that the novel usability framework can be successfully applied to the original study rather than to retrospective data. The efficiency analysis showed that patients with different levels of disability, with or without a caregiver, can successfully operate BCI for a prolonged period of time, getting a reasonable quality EEG signal. Effectiveness analysis showed that 40% of patients achieved clinically significant relief of pain,. According to the average score from QUESQ, user satisfaction was high. An important finding relevant for designers of wearable BCI technology is that there seems to be no significant stigma to using wearable EEG device in public.

Results from the interviews and observation analysis provide useful information regarding future improvements in NFB software application. While the current hardware solution was acceptable by most of participants, observation and interviews identified the need for dedicated hardware designed to wirelessly record from the central area of the cortex covered by hair, using non gel electrodes. This would also be relevant for a range of BCI applications for stroke and other patient groups.

Due to the pragmatic, uncontrolled nature of the study it was not possible to test for placebo effects, through there was evidence of correlation between selective modulation of brain activity and reduction in pain. EEG recording was limited to a single electrode to reduce donning time. Only half of the participants completed the trial, in most cases due to health issues and other reasons not directly related to NFB.

Results of the study are relevant for developers of BCI applications working with the SCI population, including assistive and rehabilitative BCI technology.

## Data Availability

Raw EEG data are available from the authors on reasonable request.

## Abbreviations

ASIA: American Spinal Injury Association
BCI: Brain Computer Interface
CNP: Central Neuropathic Pain
EEG: Electroencephalography
GUI: Graphical User Interface
NFB: Neurofeedback
PSD: Power Spectrum Density
QUESQ: Quebec User Evaluation of Satisfaction Questionnaire
rTMS: repetitive Transcranial Magnetic Stimulation
SCI: Spinal Cord Injury
tDCS: transcranial Direct Current Stimulation
VNS: Visual Numerical Scale
